# Analysis of the COVID-19 vaccine willingness and hesitancy among parents of healthy children aged 6 months–4 years: a cross-sectional survey in Italy

**DOI:** 10.3389/fpubh.2023.1241514

**Published:** 2023-10-24

**Authors:** Mario Postiglione, Grazia Miraglia del Giudice, Giorgia Della Polla, Italo Francesco Angelillo

**Affiliations:** ^1^Department of Experimental Medicine, University of Campania “Luigi Vanvitelli”, Naples, Italy; ^2^Department of Public Health and Laboratory Services, Teaching Hospital of the University of Campania “Luigi Vanvitelli”, Naples, Italy

**Keywords:** children, COVID-19, hesitancy, Italy, vaccination

## Abstract

**Introduction:**

In Italy, since December 2022, the COVID-19 vaccination has been extended to children aged 6 months–4 years with conditions of fragility and to those healthy at the request of the parent. The purposes of the cross-sectional survey were to determine the willingness and hesitancy of the parents/guardians to have their healthy children vaccinated against COVID-19.

**Methods:**

The survey was performed among 389 parents/guardians with a child aged 6 months–4 years randomly selected from seven kindergartens and eight nursery schools in the geographic area of Naples, Italy.

**Results:**

Only 10.5% were very concerned about the risk of infection, and the mean values regarding the perceived utility and safety of the COVID-19 vaccine were 3.3 and 3.2, respectively. Only 13.7% of participants were willing to consent to vaccinate the selected child against COVID-19, while 20.1% were uncertain and 66.2% did not intend. Parents/guardians of older children, those who received information about the COVID-19 vaccine from physicians or pediatricians, those who believed that the COVID-19 vaccine was useful, and those with lower hesitancy regarding the COVID-19 vaccine were more willing to vaccinate their child. The mean Parent Attitudes About Childhood Vaccines (PACV-5) score was 5.6, with 33.1% of respondents who were identified as highly hesitant toward COVID-19 vaccination (score ≥ 7). Parents/guardians with a lower perceived safety of the COVID-19 vaccine were more likely to be highly hesitant.

**Discussion:**

The findings reveal the need to improve community-based education campaigns and effective promotion of the COVID-19 vaccination to increase willingness and address parental safety concerns.

## Introduction

It is well-established that the COVID-19 vaccines are safe and effective in protecting against the SARS-CoV-2 infection and are likely one of the most important public health interventions for reducing the spread of the infection and the burden of the disease (1, 2). The population-level benefits of this vaccination are also important when delivered to a pediatric population relative to the older adult population due to the crucial role that children play in the transmission of SARS-CoV-2. In Italy, there were more than 785,000 cases among children between 6 months and 4 years and over 14,000 hospitalizations, especially in those aged 0–2 years (3). In Italy, the COVID-19 vaccination campaign for children aged 5 to 11 years began in December 2021, and currently, since December 2022, the Pfizer BioNTech COVID-19 vaccine is recommended for children aged 6 months to under 5 years of age in three separate doses for those with conditions of fragility and also for children who do not present such conditions at the request of the parent (4). However, despite this recommendation, the vaccination campaign in this age group has not yet been initiated.

Prior studies that assessed parental acceptance of the COVID-19 vaccination for their children in several countries reported refusal or delay with concerns about the safety and effectiveness of the vaccines (5–8). However, only a few studies have been specifically performed to examine this phenomenon among parents of children aged 6 months–4 years (9–11). Since parents play a dominant role in vaccination among children and adolescents, understanding their willingness and hesitancy to vaccinate their healthy children aged 6 months–4 years and the associated predictors is a vital step to better design and implement an effective vaccine information and communication strategy in order to achieve the highest possible coverage. Thus, to address this research gap, this cross-sectional survey was undertaken to determine the willingness and the hesitancy of parents in Italy to have their healthy children aged 6 months–4 years vaccinated against COVID-19 and to determine the role of different factors in such willingness and hesitancy.

## Materials and methods

### Study setting, participants, and recruitment

Data for this cross-sectional survey were part of a broader project seeking to evaluate perceptions and behaviors toward COVID-19 vaccination in different groups in southern Italy (12–19). Data collection took place from January to March 2023 in seven public kindergartens and eight public nursery schools randomly selected in the geographic area of Naples, the southern part of Italy. The parents/guardians aged ≥18 years, one per child, of all the healthy children aged 6 months–4 years were included in the survey. Parents/guardians who had more than one age-eligible child were told to respond with regard to the child with the less recent birthday.

The sample size calculation was performed according to the primary outcome “parents’ willingness to vaccinate their children.” Assuming a true willingness of 50%, a 5% type one error, and a confidence interval of 95%, a total sample size of 385 parents/guardians is needed.

### Data collection

Before initiating the survey, the heads of the selected kindergartens and nursery schools were contacted by the research team for the approval of the survey participation. After the approval, the research team, with the collaboration of the teachers, handed out to the parent/guardian in each kindergarten and nursery school an envelope with a letter of invitation to participate, an informed consent form, a questionnaire, and two envelopes to return the questionnaire and the signed informed consent for survey participation. In the letter were reported the survey aims and procedures, that all data collected would be kept confidential and anonymous, that no identifiers or personal information were included, that participation was voluntary, and that they could withdraw at any stage without providing a reason. To maximize the response rate, the reminder strategy included several follow-ups through active contact with the teachers to encourage participation and further collect data from the non-respondents. There was no participation incentive.

This research was approved by the Ethics Committee of the Teaching Hospital of the University of Campania “Luigi Vanvitelli.”

### Questionnaire

For this survey, a new questionnaire has been developed based on similar previous instruments used by some of us among different populations (14–19). The self-administered questionnaire, uploaded as Supplementary Material, was structured in three sections with 31 questions and took the parents approximately 10 min to complete. Internal consistency was calculated with Cronbach’s α.

In the first section, the interviewee reported socio-demographic and health-status characteristics of themselves, including age, gender, marital status, educational level, occupation, number of children, number of household members, previous SARS-CoV-2 infection and vaccination against COVID-19 in the household members, and their selected child, including age, gender, and having been infected by SARS-CoV-2 infection. The second section investigated attitudes around SARS-CoV-2 infection and the COVID-19 vaccine with four questions regarding the perceived risk that the selected child could be infected, the belief that COVID-19 is a serious disease, and the perceived utility and safety of the vaccine. Each question was scored on a 10-point Likert scale, with higher scores corresponding to a better attitude. Participants were also asked if they would vaccinate their child against COVID-19, and the response options were “yes,” “no,” and “uncertain.” The reason(s) why, why not, or uncertainty participants would vaccinate their child were also asked with the possibility to choose one/more predefined answers. Parents’ hesitancy regarding the COVID-19 vaccine was assessed through the 5-item version of the 15-item Parent Attitudes About Childhood Vaccines (PACV) Survey Tool, with 5-point Likert categorical responses (strongly disagree, disagree, uncertain, agree, strongly agree) (20, 21). A score of 0–2 was given to each of the five items, with non-hesitant responses getting a score of 0, responses of “not sure” getting a score of 1, and hesitant responses getting a score of 2. PACV-5 total scores were afterward categorized as low (0–4), moderate (5–6), and high hesitant (7–10). The third section queried the sources of information about the COVID-19 vaccine for children aged between 6 months and 4 years from a list of eight options and whether they would like to receive additional information.

### Statistical analysis

First, descriptive statistics were used to determine the distributions of all categorical and continuous variables. Categorical variables were expressed herein as numbers and proportions, and those continuous variables were expressed as means and standard deviations. Second, the chi-square test and the Student’s t-test were used to determine the relationship between the different outcomes of interest and each of the explanatory categorical and continuous variables, respectively. Third, variables with a value of *p* ≤0.25 in the univariate analysis were included in two multivariate logistic regression models constructed to assess the independent predictors of the following outcomes of interest: parents’ willingness to vaccinate their children aged 6 months–4 years against COVID-19 (no/do not know; yes) (Model 1); parents’ high hesitancy regarding the COVID-19 vaccine for their children aged 6 months–4 years (PACV-5 scores 1–6; PACV-5 scores 7–10) (Model 2). The following independent variables of interest regarding the respondent parent/guardian were included in the models: gender (male; female), age, in years (continuous), marital status (unmarried/separated/divorced/widowed; married/cohabited with a partner), baccalaureate/graduate degree (no; yes), number of children in household (1; >1), number of cohabitants (continuous), having been infected by SARS-CoV-2 (no; yes), at least one parent/guardian with one chronic medical condition (no; yes), and at least one adult cohabitant who has been vaccinated against COVID-19 (no; yes). The following independent variables regarding the selected child aged 6 months–4 years were also included: age, in years (<2; 2; > 2), gender (male; female), having been infected by SARS-CoV-2 infection (no; yes), belief that COVID-19 is a serious disease (continuous), perceived risk of getting SARS-CoV-2 infection (continuous), perceived utility of the COVID-19 vaccine (continuous), perceived safety of the COVID-19 vaccine (continuous), and sources of information about the COVID-19 vaccine (none; pediatricians or physicians; others). The variable parents’ high hesitancy regarding the COVID-19 vaccine for their children aged 6 months–4 years (PACV-5 scores 1–6; PACV-5 scores 7–10) was also included in Model 1. The adjusted odds ratio (OR) and the corresponding 95% confidence interval (CI) for the factors independently associated with the outcomes of interest have been reported. For all analyses, two-tailed tests were used, and a value of *p* equal to or less than 0.05 was considered statistically significant. All statistical analyses were performed using STATA software version 17.

## Results

The internal reliability of the questionnaire, measured with Cronbach’s α, was equal to 0.95. Of the 760 parents/guardians, the survey had a response rate of 51.2%. The main characteristics of parents/guardians and children are summarized in Table 1. The majority were women, the average age was 36.4 years, 91.6% were married or cohabitants, 43.6% held a university degree, 8.1% had at least one chronic medical condition, 67.4% had been infected by SARS-CoV-2, and more than 90% had received at least one shot of the COVID-19 vaccine. The selected child’s mean age was 2.6 years, 50.5% were girls, and 49.7% had been infected by SARS-CoV-2.

**Table 1:** Socio-demographic and general characteristics of the study population.

Characteristics	*N*	%
Parent/Guardian		
Age, years	36.4 ± 6.1 (23–79)^*^	
Sex		
Females	341	88.6
Males	44	11.4
Partnership status		
Married/living with a partner	348	91.6
Unmarried	32	8.4
Educational level		
High school degree or less	217	56.4
Baccalaureate/graduate degree	168	43.6
Employment status		
Unemployed	170	45.6
Employed	203	54.4
At least one parent with one chronic medical condition		
No	365	95.3
Yes	18	8.1
Having had a personal history of SARS-CoV-2 infection		
No	126	32.6
Yes	261	67.4
Having had at least one shot of COVID-19 vaccine		
No	24	6.4
Yes	351	93.6
Having more than one child		
No	135	35.3
Yes	248	64.7
Selected child		
Age, years	2.6 ± 1.1°	
6 months–1 year	66	17.5
2	92	24.5
3–4	218	58
Sex		
Females	188	50.5
Male	184	49.5
Having been infected by SARS-CoV-2		
No	193	50.3
Yes	191	49.7

Number for each item may not add up to the total number of the study population due to missing values. ^*^Mean ± standard deviation (range). ° Mean ± standard deviation.

With regard to attitudes about COVID-19 disease and vaccination, measured with a 10-point Likert scale, the mean values of the concern that COVID-19 was a serious disease and that their children may be infected by SARS-CoV-2 were 5.1 and 5.3, respectively. Only 10.5% were very concerned about the risk of infection, with a value of 10. The mean values regarding the perceived utility and safety of the COVID-19 vaccine were 3.3 and 3.2, respectively, and a third (34.2%) indicated that they were not concerned about side effects. Only 13.7% of participants were willing to consent to vaccinate the selected child against COVID-19, while 20.1% were uncertain and 66.2% did not intend. Results from the multivariate logistic regression analysis performed to determine the variables associated with the two outcomes of interest are summarized in Table 2. Parents/guardians of older age (OR = 1.16; 95% CI = 1.05–1.28), those who received information about the COVID-19 vaccine from physicians or pediatricians (OR = 15.31; 95% CI = 2.97–78.83), those who believed that the COVID-19 vaccine was useful (OR = 2.31; 95% CI = 1.62–3.27), and those with lower vaccine hesitancy regarding the COVID-19 vaccine (OR = 0.12; 95% CI = 0.01–0.98) were more willing to vaccinate their selected child aged 6 months–4 years against COVID-19 (Model 1). The three most common reasons provided behind their willingness were that the vaccination could protect their children (64.8%), trust in vaccinations (51.8%), and efficacy of the vaccine (46.3%). The most frequent perceived reasons for parents/guardians who did not intend or were uncertain about vaccinating their children were concerns about the safety of the vaccine (67.5 and 74.7%) and that the vaccine would not prevent the infection (40.2 and 33.3%).

**Table 2:** Multivariate logistic regression analysis results examine the determinants of the different outcomes of interest.

Variable	OR	SE	95% CI	*p*
*Model 1*. Parents’ willingness to vaccinate their children aged 6 months–4 years against COVID-19 Log-likelihood = −48.12, χ^2^ = 176.73 (9 df), *p* < 0.0001				
Higher perceived utility of the COVID-19 vaccine	2.31	0.41	1.62–3.27	<0.001
Sources of information				
None	1.00^*^			
Pediatricians/Physicians	15.31	12.81	2.97–78.83	0.001
Others	5.48	4.87	0.95–31.33	0.056
Older parents/guardians	1.16	0.05	1.05–1.28	0.003
Lower parental vaccine hesitancy against COVID-19	0.12	0.13	0.01–0.98	0.049
Having more than one child	0.31	0.19	0.09–1.05	0.059
Higher perceived safety of the COVID-19 vaccine	1.25	0.21	0.91–1.71	0.172
Lower perceived risk for their children of getting SARS-CoV-2 infection	0.83	0.12	0.63–1.10	0.203
Having their child being infected by SARS-CoV-2 infection	1.72	0.96	0.58–5.13	0.329
*Model 2*. Parents’ high hesitancy regarding the COVID-19 vaccine for their children aged 6 months–4 years Log-likelihood = −183.32, χ^2^ = 42.21 (6 df), *p* < 0.0001				
Lower perceived safety of the COVID-19 vaccine	0.81	0.08	0.65–0.98	0.035
Lower perceived utility of the COVID-19 vaccine	0.86	0.09	0.71–1.05	0.138
Child age, in years				
6 months–1 year	1.00^*^			
2	1.58	0.67	0.69–3.63	0.278
3–4	1.73	0.66	0.82–3.65	0.149
Sources of information				
None	1.00^*^			
Pediatricians/Physicians	0.73	0.21	0.42–1.26	0.265
Higher perceived risk for their children of getting SARS-CoV-2 infection	1.06	0.06	0.96–1.18	0.230

^*^Reference category.

The mean score of the PACV-5 was 5.6, with 33.1% of respondents who were identified as high hesitant toward COVID-19 vaccination (score ≥ 7), 37.7% as moderate hesitant (scores 4–6), and 29.2% as low hesitant (scores 0–3). Parents/guardians with a PACV-5 score of 0 and 10 were 1.9% and 5.4%, respectively. As shown in Table 3, one-fourth (25.5%) strongly agreed/agreed that children get too many shots, 62% strongly agreed/agreed that it is better that children get fewer vaccines at the same time, and 51.4% strongly disagreed/disagreed that it is better for the child to develop immunity by getting sick than to get a shot. Two-thirds (63.6%) considered themselves to be hesitant about giving their children a dose of the COVID-19 vaccine. Only 14.5% trusted the information received on the issue of the COVID-19 vaccine. Multivariate logistic regression analysis was developed to estimate the predictors of parental COVID-19 vaccine high hesitancy for children aged 6 months–4 years, and the results revealed that only one variable was significantly associated with this outcome. Parents/guardians with a lower perceived safety of the COVID-19 vaccine (OR = 0.81; 95% CI = 0.65–0.98) were more likely to be highly hesitant (Model 2 in Table 2).

**Table 3:** Descriptive characteristics of PACV-5 survey tool about COVID-19 vaccine.

Item	Parent/Guardian response	*N* (%)
Children get more shots than are good for them	Strongly agree/Agree Strongly disagree/Disagree Not sure	97 (25.5) 177 (46.5) 107 (28)
It is better for my child to develop immunity by getting sick than to get a shot	Strongly agree/Agree Strongly disagree/Disagree Not sure	70 (18.3) 197 (51.4) 116 (30.3)
It is better for children to get fewer shots at the same time	Strongly agree/Agree Strongly disagree/Disagree Not sure	237 (62) 50 (13.1) 95 (24.9)
Overall, how hesitant about the COVID-19 vaccine for your child would you consider yourself to be?	Very hesitant/ Somewhat hesitant Not hesitant at all/Not too hesitant Not sure	239 (63.6) 66 (17.5) 71 (18.9)
I trust the information I receive about the COVID-19 vaccine	Strongly agree/Agree Strongly disagree/Disagree Not sure	55 (14.5) 154 (40.5) 171 (45)

Number for each item may not add up to the total number of the study population due to missing values.

Almost three-quarters of respondents (70%) reported that they sought information on the COVID-19 vaccination for children aged 6 months to 4 years. The most reliable sources of information were physicians (33.8%) and institutional websites (29.7%), followed by the Internet (26.7%) and mass media (24.5%). More than half (42.3%) stated that they needed additional information.

## Discussion

To the best of our knowledge, this survey is the first to look at the willingness and hesitancy toward the vaccine against COVID-19 among parents of children aged 6 months–4 years in Italy. The present findings add to the global literature on COVID-19 vaccination intentions and hesitancy among parents.

When assessing the parents’ willingness to vaccinate their children against COVID-19, only 14.4% intended to vaccinate. This value was considerably lower than the intention observed with a large variation among parents of similar-aged healthy children in other studies in different countries, like for example in Australia (77.2%) (22), Brazil (73%) (23), United States (51.5%) (24), Ireland (50.6%) (25), Thailand (45.1%) (9), and Canada (41.9–45.4%) (26). A possible explanation for the very low willingness toward COVID-19 vaccination is the fact that at the moment of this survey, the campaign for children aged 6 months–4 years had not yet started in Italy, and, therefore, parents are looking for more information before exposing their children. Moreover, the value was also lower than the 29.5% observed in the same geographic area among parents of similar-aged children with chronic medical conditions (27). The reasons cited by the participants who were willing to receive the COVID-19 vaccination included the protection of their children, trust in vaccinations, and the efficacy of the vaccine. Concerns about vaccine safety were shared by more than two-thirds of parents who did not intend or were uncertain to vaccinate their children. These agree with the existing literature, in which the most frequently cited reasons for why parents or adults accept this vaccination are to protect themselves (28) or their children (29), whereas for refusal the perceived risk of adverse effects (30). Therefore, it is important that healthcare workers, mainly pediatricians and public health professionals, discuss the parent’s concerns and use tailored information in their vaccine discussions. Deeper healthcare workers–parent vaccine interactions are strongly needed since they may reduce unwillingness and hesitancy associated with a consequent increase in vaccination coverage.

Another important finding of the current survey is the considerably high frequency of participants who were either high hesitant (33.1%) or moderate hesitant (37.7%) to COVID-19 vaccination for their children assessed with the PACV-5 instrument. A multivariate logistic regression model revealed that only one factor played a significant role in determining the high hesitancy of the parental vaccine. Respondents who did not believe that the vaccine was safe were more likely to be highly hesitant. Again, health education and health promotion efforts were needed, involving parents in the COVID-19 vaccination campaign to educate them about vaccine safety and efficacy and ultimately improve their acceptance (27, 31).

A clearer identification of the parents’ socio-demographic and general characteristics significantly related to their unwillingness or high hesitancy about the COVID-19 vaccine for their healthy children aged 6 months–4 years may be useful for designing tailored interventions targeted to effectively influence parental decision-making regarding this vaccination. It has been observed that parental age was a significant predictor of the respondents’ willingness to vaccinate their children. Parents of older age had higher odds of being willing to vaccinate their children than those of younger age. The association with age probably reflects the fact that older parents have had more previous opportunities to be vaccinated during their lifetime and, therefore, they have a more positive attitude toward this vaccination (32, 33).

It was worth noting that parents in this survey indicated physicians and governmental institutions as the most frequently used sources of information on COVID-19 vaccination for their children aged 6 months–4 years. The multivariate logistic regression analysis highlighted the important role that physicians have in vaccine decision-making since parents who inform themselves from this source are more likely to be willing to vaccinate their children than those who do not have acquired information or have used other sources. This finding has already been well-documented in prior studies concerned with COVID-19 vaccination among different groups of individuals conducted in this geographic area (16, 17) and elsewhere (34, 35) that found physicians to have a key role in positively influencing knowledge and vaccine decision-making. This may be explained by the fact that parents may prefer to vaccinate their child with their physicians, with whom they have had a longer relationship and time to develop trust and understanding. Since the source of information was linked with the intention to vaccinate, physicians, as with all other healthcare workers, need to be used as a tool to address parents’ concerns and to disseminate and communicate accurate and appropriate COVID-19 vaccine information in order to improve their confidence in the safety of this vaccination. Moreover, it has been observed that many parents have encountered information through social networks and internet sources, which may have a negative impact on their understanding and decision toward this vaccination for their children. This is of concern because previous literature related to vaccines pointed out that social networks and the Internet are sources of vaccine misinformation and knowledge gaps by determining unwillingness, hesitancy, and low uptake (36–38).

Some methodological limitations should be considered in the interpretation of the findings of this survey. First, it had a cross-sectional design, meaning that conclusions cannot be drawn regarding the causal relationships between parental willingness and vaccine hesitancy and the different characteristics. Second, the parents were selected in a geographic area in southern Italy, and, therefore, the findings might not be totally generalizable to other areas of the country. Third, the survey relied on self-reported characteristics of parent vaccine intentions, which may lead to a social desirability bias that might potentially affect the accuracy of the data. However, in order to minimize this bias, no particular personal or sensitive data has been collected, and all responses were anonymous.

To conclude, the findings of this survey among parents in Italy revealed a very low level of acceptance of the COVID-19 vaccine for their children aged from 6 months to 4 years, as only 13.7% intended to vaccinate them and one-third were highly hesitant. Almost two-thirds of the sample were concerned about COVID-19 vaccine safety and potential side effects, and almost one-third have never sought information on this topic. There is great room for improvement with community-based education campaigns and effective promotion, involving different healthcare workers, of the COVID-19 vaccination to increase willingness and address parental safety concerns.

## Data availability statement

The raw data supporting the conclusions of this article will be made available by the authors, without undue reservation.

## Ethics statement

The study involving humans was approved by Ethics Committee of the Teaching Hospital of the University of Campania “Luigi Vanvitelli.” The study was conducted in accordance with the local legislation and institutional requirements. The participants provided their written informed consent to participate in this study.

## Author contributions

MP, GMdG, and GDP participated in the conception and design of the study, contributed to the data collection, data analysis, and interpretation. IFA was principal investigator, designed the study, and responsible for the statistical analysis and interpretation, and wrote the manuscript. All authors contributed to the article and approved the submitted version.
