# Supplementary material for: Analysis of the COVID-19 vaccine willingness and hesitancy among parents of healthy children aged 6 months–4 years: a cross-sectional survey in Italy
Source: Front Public Health. 2023 Oct 24;11:1241514. doi: 10.3389/fpubh.2023.1241514 (PMC10628319; doi:10.3389/fpubh.2023.1241514)
Supplement: Supplementary file 1 [file Data_Sheet_1.DOCX]

Questionnaire. **Supplementary Material**

**A. SOCIO-DEMOGRAPHIC AND ANAMNESTIC CHARACTERISTICS**

**This section is designed to gather information about your socio-demographic and anamnestic characteristics**

**A1.** How old were you on your last birthday?________

**A2.**What is your gender? □ Male □ Female

**A3.** What is your marital status? □ Married □ Cohabiting □ Other (*please specifiy)____________*

**A4.**What is your highest educational level? □ None □ Primary school □ High school □ Baccalaurate □ Graduate

**A5.** What is your partner’s highest educational level? □ None □ Primary school □ High school □ Baccalaurate □ Graduate

**A6.** What is your occupation?**________________**

**A7.** Do you have any chronic medical condition? □ No □ Yes (*please specifiy, more than one disease is allowed*)**_________________**

**A8.** Do your parents/family members have any chronic medical condition? □ No □ Yes

**A9.** Have you ever been infected by SARS-CoV-2? □ No □ Yes

**A10.** Has any parent /family member been infected by SARS-CoV-2? □ No □ Yes

**A11.** Have you been vaccinated against COVID-19? □ No □ Yes, how many doses? (*please specify the number*) _______

**A12.** Has any parent/family member been vaccinated against COVID-19?

□ No □ Yes, *please indicate age and number of doses for each*

| ***Age*** | ***Number of doses*** | ***Age*** | ***Number of doses*** | ***Age*** | ***Number of doses*** |
| --- | --- | --- | --- | --- | --- |
|  |  |  |  |  |  |
|  |  |  |  |  |  |

**A13.** How many children do you have? (*please specify the number*) ______

**A14***.* How old are your children? (*please specify in years or in months if <1 year*)

First-born_____ Second-born_____ Third-born_____ Fourth-born_____

***The following questions refer to your youngest child aged 6 months-4 years***

**A15.** What is your child’s birth order? □ First □ Second □ Third □ Fourth □ Other *(please specify)* _____________

**A16.** What is your child’s gender? □ Male □ Female

**A17.** Has your child been infected by SARS-CoV-2? □ No □ Yes

**B. ATTITUDES**

**This section is designed to explore your attitudes towards SARS-CoV-2 infection and its vaccination for your youngest child aged 6 months-4 years**

**B1.** On a scale from 1 to10, how serious do you consider the SARS-CoV-2 infection for your child? *(1 indicates that you do not consider it serious at all, 10 if you consider it a very serious disease)*

**Not serious** 1 2 3 4 5 6 7 8 9 10 **Very serious**

**B2.** On a scale from 1 to 10, how much do you perceive your child at risk of getting the SARS-CoV-2 infection? *(1 indicates that you do not consider your child at risk at all, 10 if you consider your child very much at risk)*

**Not at risk** 1 2 3 4 5 6 7 8 9 10 **Very much at risk**

**B3.** On a scale from 1 to 10, how useful do you consider the COVID-19 vaccination for your child? (*1 indicates that you do not consider it useful at all, 10 if you consider it very useful)*

**Not useful** 1 2 3 4 5 6 7 8 9 10 **Very useful**

**B4.** On a scale from 1 to 10, how safe do you consider the COVID-19 vaccination for your child? (*1 indicates that you do not consider it safe at all, 10 if you consider it very safe)*

**Not safe** 1 2 3 4 5 6 7 8 9 10 **Very safe**

**B5.** Have you ever delayed having your child get a shot for reasons other than illness or allergy?

□No □Yes □Do not know

**B6.** Have you ever decided not to have your child get a shot for reasons other than illness or allergy?

□No □Yes □Do not know

**B7.** Children get more shots than are good for them.

| □Strongly disagree | □Disagree | □Not sure | □Agree | □Strongly agree |
| --- | --- | --- | --- | --- |

**B8.** It is better for my child to develop immunity by getting sick than to get a shot.

| □Strongly disagree | □Disagree | □Not sure | □Agree | □Strongly agree |
| --- | --- | --- | --- | --- |

**B9.** It is better for children to get fewer vaccines at the same time.

| □Strongly disagree | □Disagree | □Not sure | □Agree | □Strongly agree |
| --- | --- | --- | --- | --- |

**B10.** Overall, how hesitant about COVID-19 vaccine for your child would you consider yourself to be?

| □Very hesitant | □Somewhat hesitant | □Not sure | □Not too hesitant | □Not hesitant at all |
| --- | --- | --- | --- | --- |

**B11.** I trust the information I receive about COVID-19 vaccination for children.

| □Strongly disagree | □Disagree | □Not sure | □Agree | □Strongly agree |
| --- | --- | --- | --- | --- |

**B12.** Would you vaccinate your child against COVID-19?

□Yes □No □Uncertain

| Why would you vaccinate your child?  *(more than one answer is allowed)* | Why would you not vaccinate your child?  *(more than one answer is allowed)* | Why are you uncertain to vaccinate your child?  *(more than one answer is allowed)* |
| --- | --- | --- |
| □To prevent the onset of COVID-19 | □COVID-19 vaccination does not prevent the onset of the disease | □I am not sure if COVID-19 vaccination prevents the onset of the disease |
| □I trust COVID-19 vaccination | □I do not trust COVID-19 vaccination | □I am not sure if I trust COVID-19 vaccination |
| □To protect my family members from getting COVID-19 | □ The vaccination is not useful to protect my family members from getting COVID-19 | □I am not sure if the vaccination is useful to protect my family members from getting COVID-19 |
| □I consider my child at risk of getting COVID-19 | □I do not consider my child at risk of getting COVID-19 | □I am not sure if my child is at risk from getting COVID-19 |
| □ Recommended by a pediatrician | □ Not recommended by a pediatrician | □ Not recommended by a pediatrician |
| □COVID-19 vaccination is safe | □ Concern for side effects of the COVID-19 vaccination | □Concern for side effects of the COVID-19 vaccination |
| □COVID-19 vaccination is effective | □COVID-19 vaccination is not effective | □I am not sure that COVID-19 vaccination is effective |
| □COVID-19 is a serious disease for my child | □COVID-19 is not a serious disease for my child | □I am not sure that COVID-19 is a serious disease for my child |
| □Vaccination’s benefits are higher than the risks | □Vaccination’s risks are higher than the benefits | □I am not sure that vaccination’s benefits are higher than the risks |
| □I trust in vaccines | □I do not trust in vaccines | □I am not sure if I trust in vaccines |
| □To protect my child from getting COVID-19 | □I do not have information | □I do not have adequate information |
| □The information that I have received are reliable | □The information that I have received are not reliable | □I am not sure if the information that I have received are reliable |

**C. INFORMATION**

**This section is designed to explore your sources of information about the COVID-19 vaccination for your youngest child aged 6 months-4 years**

**C1.** Which sources did you use to receive information about the COVID-19 vaccination for children aged 6 months-4 years?*(more than one source is allowed)* □None

□Healthcare workers *(please specify)* _________________________ □Pediatrician □Internet □ Mass media

□Institutional organizations □ Social media (Facebook, Twitter, Instagram, YouTube, etc.) □Friends and Family

□Scientific journals □Other*(please specify)*_______________________________________________________________________

**C2.** Do you need further information about COVID-19 vaccine for children aged 6 months-4 years? □No □Yes
